# Artificial Teeth Attached to an Auroplastic Base

**Published:** 1853-10

**Authors:** 


***Artificial Teeth Attached to an Auroplastic Base.***—Mr. Edwin Truman, dentist, of London, has recently published a treatise on the application of artificial teeth upon the auroplastic principle. The base is formed of gutta percha, and the exposed surface plated with gold, by means of electro-galvanism. The author has secured to himself the exclusive right of the principle by letters patent. We publish the treatise entire, except the preface, among the selected articles of the present issue of the Journal. Whether artificial teeth mounted upon a base of this sort, will stand the test of experience, is, we think, more than questionable. We believe, therefore, that we hazard little in saying, *a priori*, that the substitution of it for gold and platina, the metals now generally employed for this purpose, will never become general.

In alluding to the use of gutta percha as a base for artificial teeth in a former number of the Journal, we stated that we believed Dr. Hill, of Norwich, Ct., was the first to test its applicability for this purpose. He informed us by letter, about four years ago, if our memory serves us correctly, that he had then made some experiments with it, which promised the most satisfactory results. We should be glad to hear from him again upon the subject, and learn to what extent his expectations had, by his subsequent experience, been realized.

